# The genome sequence of the Feathered Gothic, *Tholera decimalis* (Poda, 1761)

**DOI:** 10.12688/wellcomeopenres.19395.1

**Published:** 2023-05-05

**Authors:** Douglas Boyes, Peter W.H. Holland, University of Oxford and Wytham Woods Genome Acquisition Lab, Darwin Tree of Life Barcoding collective, Wellcome Sanger Institute Tree of Life programme, Wellcome Sanger Institute Scientific Operations: DNA Pipelines collective, Tree of Life Core Informatics collective, Darwin Tree of Life Consortium

**Affiliations:** 1 UK Centre for Ecology & Hydrology, Wallingford, England, UK; 2 University of Oxford, Oxford, England, UK

**Keywords:** Tholera decimalis, Feathered Gothic, genome sequence, chromosomal, Lepidoptera

## Abstract

We present a genome assembly from an individual female *Tholera decimalis* (the Feathered Gothic; Arthropoda; Insecta; Lepidoptera; Noctuidae). The genome sequence is 1,334.1 megabases in span. Most of the assembly is scaffolded into 31 chromosomal pseudomolecules, including the Z sex chromosome. The mitochondrial genome has also been assembled and is 15.4 kilobases in length. Gene annotation of this assembly on Ensembl identified 12,771 protein coding genes.

## Species taxonomy

Eukaryota; Metazoa; Ecdysozoa; Arthropoda; Hexapoda; Insecta; Pterygota; Neoptera; Endopterygota; Lepidoptera; Glossata; Ditrysia; Noctuoidea; Noctuidae; Hadeninae; *Tholera*; *Tholera decimalis* (Poda, 1761) (NCBI:txid988041).

## Background

The use of a network of standardised light-traps operated every night since 1970, coupled with millions of individual records collated by amateur and professional recorders, has permitted analysis of long-term trends in moth abundance. These studies indicate that over 60% of larger moth species have decreased in abundance in Britain in the past half century ( Conrad *et al.*, 2006; Fox *et al.,* 2019; Randle *et al.*, 2019). The picture for geographical distribution within Britain is more complex, with roughly equal numbers of species expanding their range or facing range contraction ( Randle *et al.*, 2019). The Feathered Gothic *Tholera decimalis* is a moth that has had mixed fortunes in this period and may provide a useful study case. The quantitative data indicate that *T. decimalis* has suffered a large decline in abundance since 1970, while its geographic range showed a large contraction followed by a more recent expansion from 2000 to 2016 ( Randle *et al.*, 2019).

Currently, *T. decimalis* is found widely across England and Wales, particularly in southern counties, but it is now absent from most of Scotland and very rare in Ireland and Northern Ireland ( National Biodiversity Data Centre, 2023; Randle *et al.*, 2019; Thompson & Nelson, 2003). The species has a patchy distribution across Eurasia with records concentrated in the Netherlands, Austria, Switzerland and Scandinavia; there are sporadic records further east through Russia to Mongolia ( GBIF Secretariat, 2023). The moth is associated with rough grassland, downland and open woodland, with adults laying eggs during August and September. The eggs overwinter before the hatched larvae feed on grasses between March and July ( Stokoe, 1948). The adult moth has bold white markings along the wing veins, streaked over ornately patterned rich brown forewings. Males have very pronounced bipectinate (feathered) antennae; this feature, not seen in females, is likely an adaptation to accommodate increased numbers of olfactory receptors for pheromone detection. Further research is needed into the chemical biology of this species, particularly as electroantennogram recording showed no response to 30 putative pheromone components that elicit responses in related species ( Renou *et al.*, 1991).

A genome sequence for *T. decimalis* will enable studies into the biochemical basis of olfactory reception and molecular adaptations for grass feeding, and may facilitate future research into the biological factors affecting responses to environmental and land use changes.

### Genome sequence report

**Figure 1.**
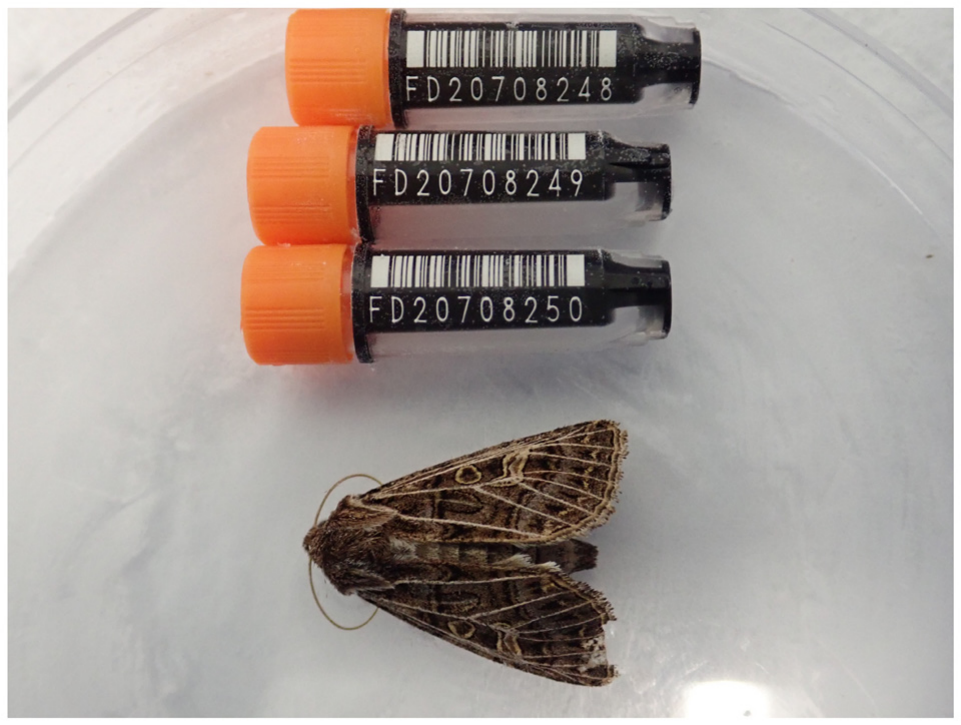
Photograph of the *Tholera decimalis* (ilThoDeci1) specimen used for genome sequencing.

The genome was sequenced from one female *Tholera decimalis* ( Figure 1) collected from Wytham Woods, Oxfordshire, UK (latitude 51.77, longitude –1.34). A total of 45-fold coverage in Pacific Biosciences single-molecule HiFi long reads and 29-fold coverage in 10X Genomics read clouds were generated. Primary assembly contigs were scaffolded with chromosome conformation Hi-C data. Manual assembly curation corrected 58 missing or mis-joins and removed 22 haplotypic duplications, reducing the assembly length by 1.26% and the scaffold number by 25%.

**Figure 2.**
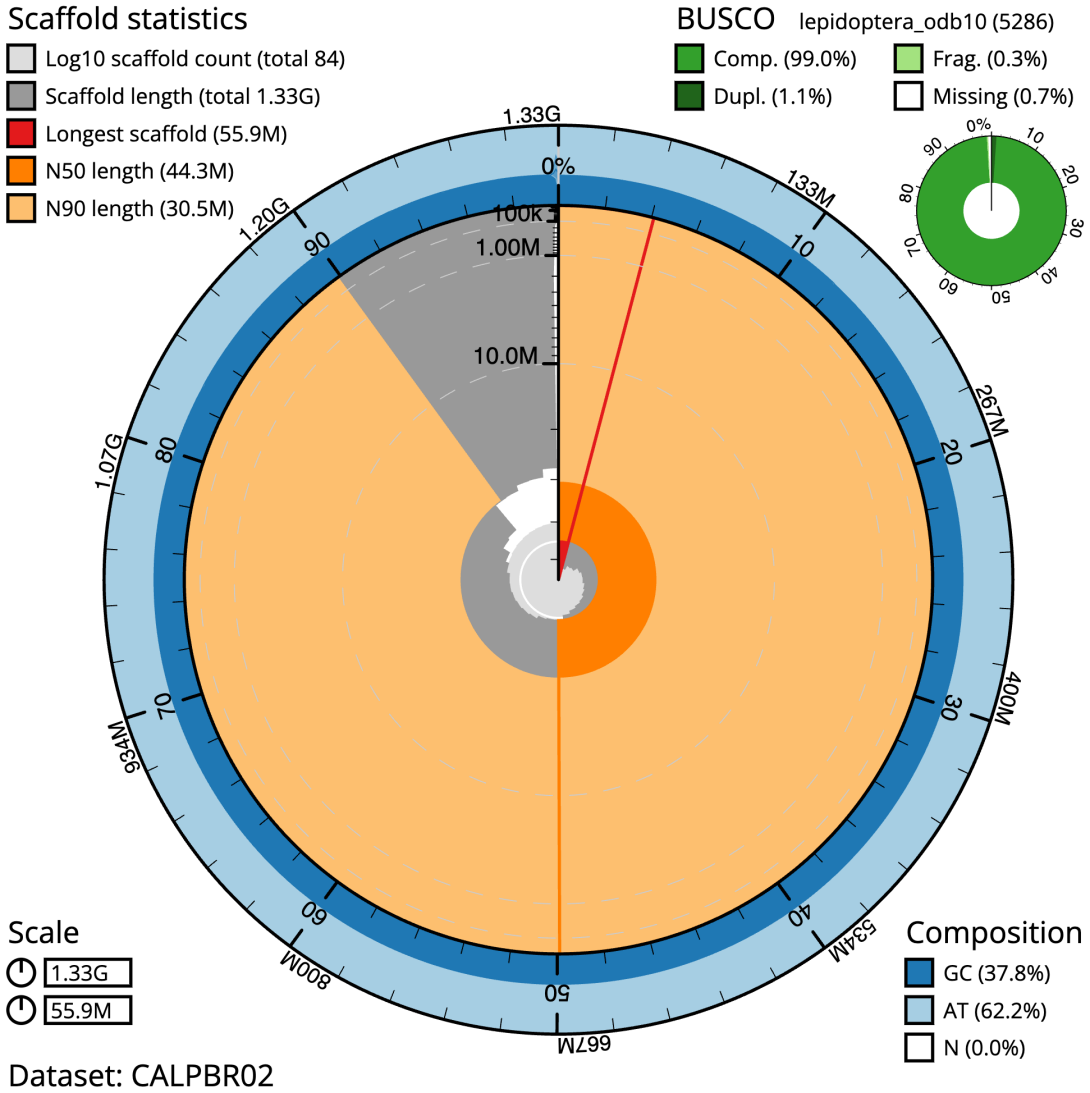
Genome assembly of *Tholera decimalis*, ilThoDeci1.2: metrics. The BlobToolKit Snailplot shows N50 metrics and BUSCO gene completeness. The main plot is divided into 1,000 size-ordered bins around the circumference with each bin representing 0.1% of the 1,334,081,899 bp assembly. The distribution of scaffold lengths is shown in dark grey with the plot radius scaled to the longest scaffold present in the assembly (55,901,591 bp, shown in red). Orange and pale-orange arcs show the N50 and N90 scaffold lengths (44,326,127 and 30,461,373 bp), respectively. The pale grey spiral shows the cumulative scaffold count on a log scale with white scale lines showing successive orders of magnitude. The blue and pale-blue area around the outside of the plot shows the distribution of GC, AT and N percentages in the same bins as the inner plot. A summary of complete, fragmented, duplicated and missing BUSCO genes in the lepidoptera_odb10 set is shown in the top right. An interactive version of this figure is available at https://blobtoolkit.genomehubs.org/view/ilThoDeci1.2/dataset/CALPBR02/snail.

The final assembly has a total length of 1,334.1 Mb in 84 sequence scaffolds with a scaffold N50 of 44.3 Mb ( Table 1). Most (99.75%) of the assembly sequence was assigned to 31 chromosomal-level scaffolds, representing 30 autosomes and the Z sex chromosome. Chromosome-scale scaffolds confirmed by the Hi-C data are named in order of size ( Figure 2– Figure 5; Table 2). While not fully phased, the assembly deposited is of one haplotype. Contigs corresponding to the second haplotype have also been deposited. The mitochondrial genome was also assembled and can be found as a contig within the multifasta file of the genome submission.

The estimated Quality Value (QV) of the final assembly is 64.6 with *k*-mer completeness of 100%, and the assembly has a BUSCO v5.3.2 completeness of 99.0% (single = 97.9%, duplicated = 1.1%), using the lepidoptera_odb10 reference set ( *n* = 5,286).

**Table 1.** Genome data for *Tholera decimalis*, ilThoDeci1.2.

Project accession data		
Assembly identifier	ilThoDeci1.2	
Species	*Tholera decimalis*	
Specimen	ilThoDeci1	
NCBI taxonomy ID	988041	
BioProject	PRJEB52581	
BioSample ID	SAMEA8603189	
Isolate information	ilThoDeci1, female: head and thorax (genome sequencing and Hi-C scaffolding); abdomen (RNA sequencing)	
Assembly metrics *		*Benchmark*
Consensus quality (QV)	64.6	*≥ 50*
*k*-mer completeness	100%	*≥ 95%*
BUSCO **	C:99.0%[S:97.9%,D:1.1%], F:0.3%,M:0.7%,n:5,286	*C ≥ 95%*
Percentage of assembly mapped to chromosomes	99.75%	*≥ 95%*
Sex chromosomes	Z chromosome	*localised homologous pairs*
Organelles	Mitochondrial genome assembled	*complete single alleles*
Raw data accessions		
PacificBiosciences SEQUEL II	ERR9709328, ERR9709329, ERR9709330, ERR9709331, ERR9709332	
10X Genomics Illumina	ERR9682493–ERR9682496	
Hi-C Illumina	ERR9682498	
PolyA RNA-Seq Illumina	ERR9682497	
**Genome assembly**		
Assembly accession	GCA_943138885.2	
*Accession of alternate haplotype*	GCA_943138005.2	
Span (Mb)	1,334.1	
Number of contigs	311	
Contig N50 length (Mb)	10.2	
Number of scaffolds	84	
Scaffold N50 length (Mb)	44.3	
Longest scaffold (Mb)	55.9	
Genome annotation		
Number of protein-coding genes	12,771	
Number of non-coding genes	1,968	
Number of gene transcripts	22,825	

* Assembly metric benchmarks are adapted from column VGP-2020 of “Table 1: Proposed standards and metrics for defining genome assembly quality” from ( Rhie *et al.*, 2021). ** BUSCO scores based on the lepidoptera_odb10 BUSCO set using v5.3.2. C = complete [S = single copy, D = duplicated], F = fragmented, M = missing, n = number of orthologues in comparison. A full set of BUSCO scores is available at https://blobtoolkit.genomehubs.org/view/ilThoDeci1.2/dataset/CALPBR02/busco.

**Figure 3.**
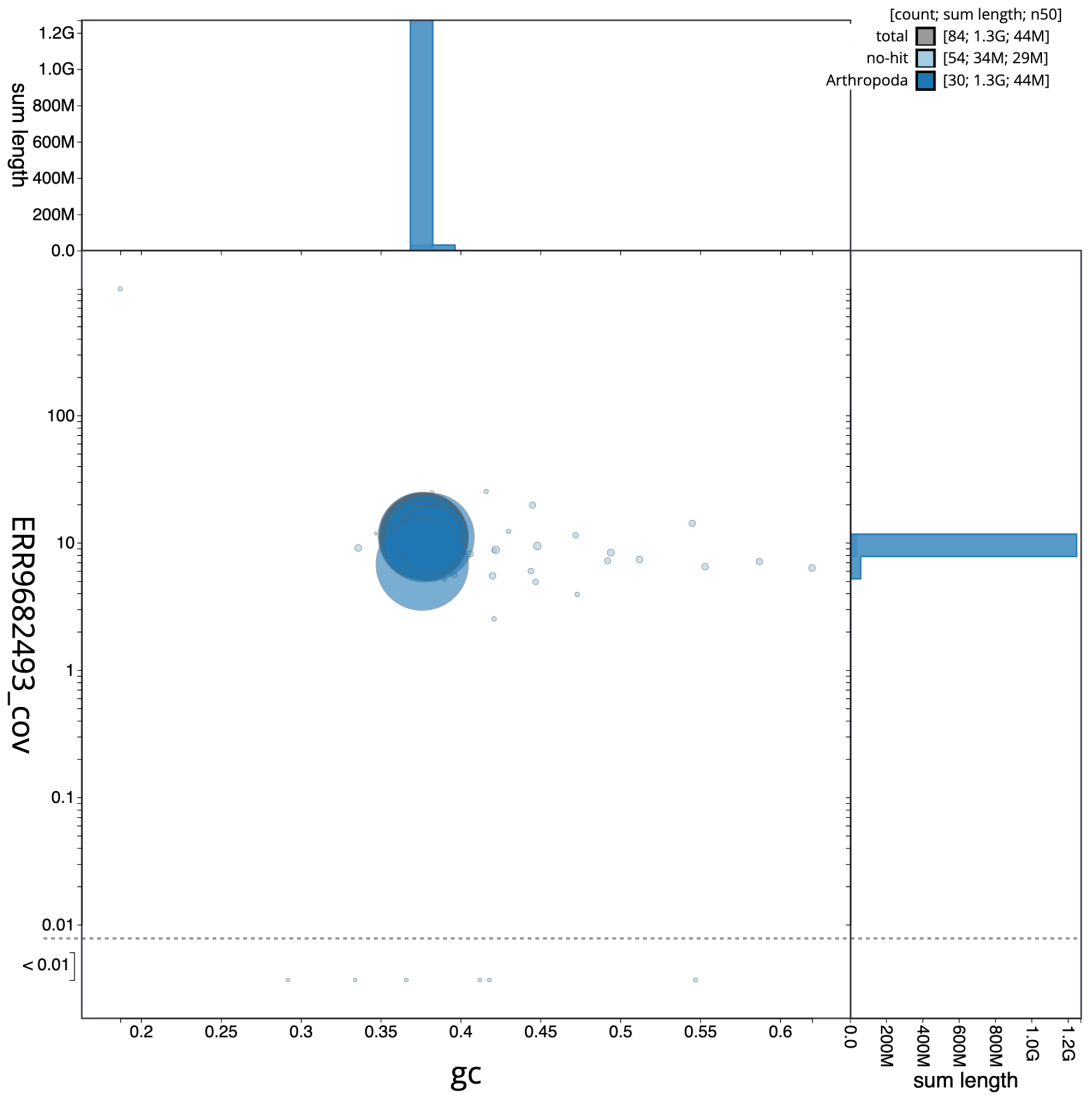
Genome assembly of *Tholera decimalis*, ilThoDeci1.2: GC coverage. BlobToolKit GC-coverage plot. Scaffolds are coloured by phylum. Circles are sized in proportion to scaffold length. Histograms show the distribution of scaffold length sum along each axis. An interactive version of this figure is available at https://blobtoolkit.genomehubs.org/view/ilThoDeci1.2/dataset/CALPBR02/blob.

**Figure 4.**
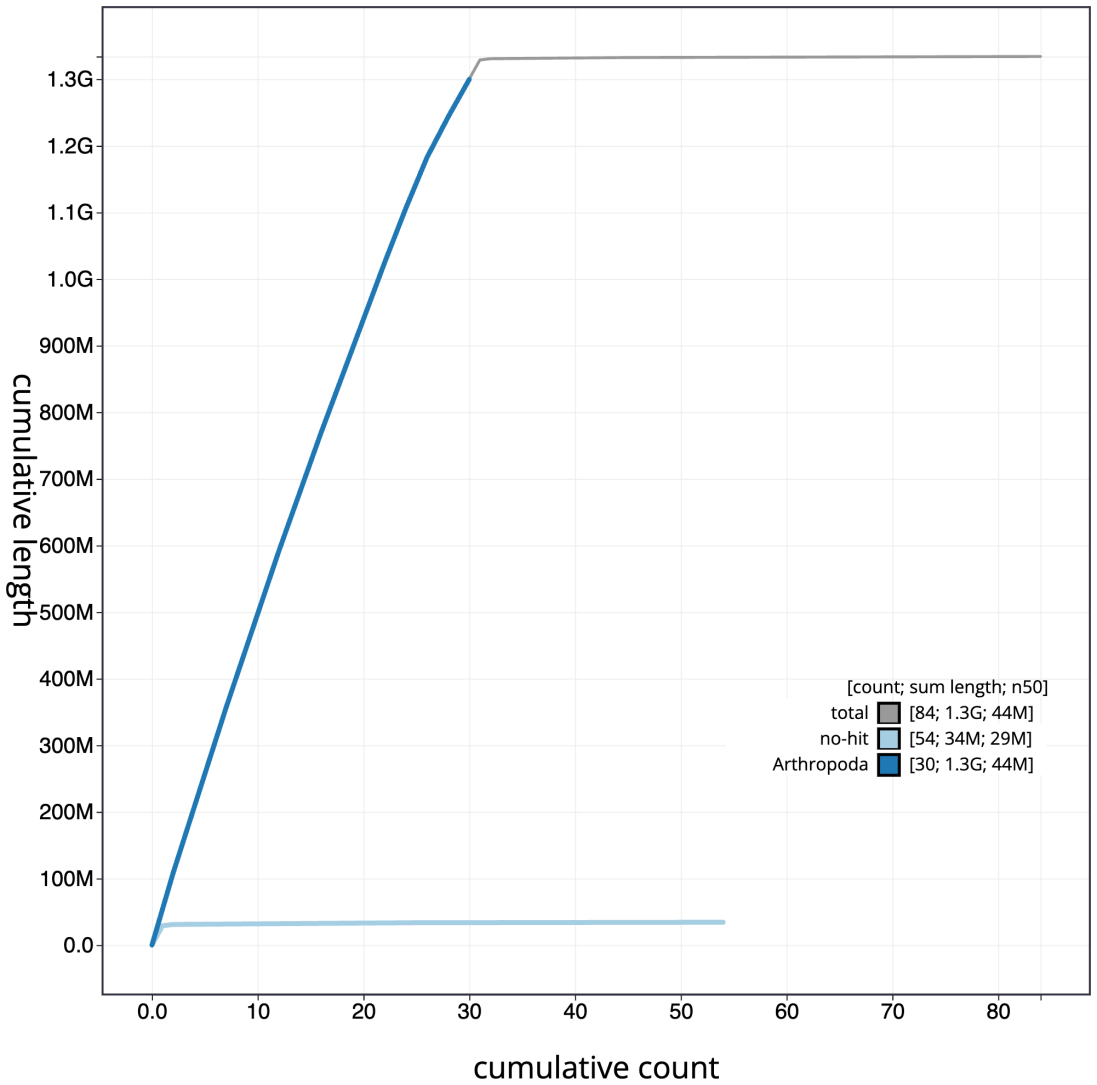
Genome assembly of *Tholera decimalis*, ilThoDeci1.2: cumulative sequence. BlobToolKit cumulative sequence plot. The grey line shows cumulative length for all scaffolds. Coloured lines show cumulative lengths of scaffolds assigned to each phylum using the buscogenes taxrule. An interactive version of this figure is available at https://blobtoolkit.genomehubs.org/view/ilThoDeci1.2/dataset/CALPBR02/cumulative.

**Figure 5.**
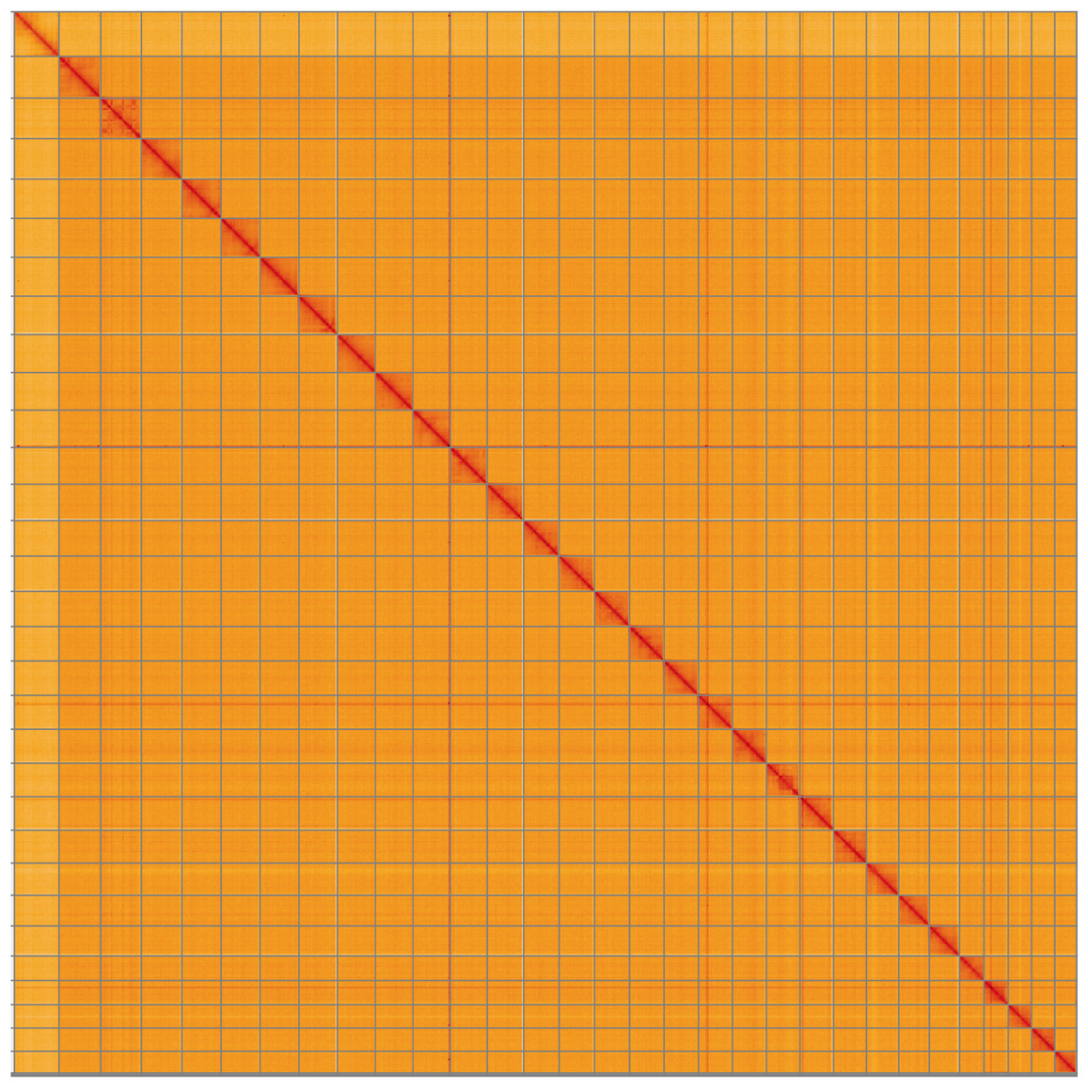
Genome assembly of *Tholera decimalis*, ilThoDeci1.2: Hi-C contact map. Hi-C contact map of the ilThoDeci1.2 assembly, visualised using HiGlass. Chromosomes are shown in order of size from left to right and top to bottom. An interactive version of this figure may be viewed at https://genome-note-higlass.tol.sanger.ac.uk/l/?d=IsQwpVaRQ36c521_VU0W0w.

**Table 2.** Chromosomal pseudomolecules in the genome assembly of *Tholera decimalis*, ilThoDeci1.

INSDC accession	Chromosome	Size (Mb)	GC%
OW964549.2	1	52.14	37.7
OW964550.2	2	50.54	37.6
OW964551.2	3	50.86	38.1
OW964552.2	4	49.27	37.6
OW964553.2	5	48.6	37.7
OW964554.2	6	48.73	37.7
OW964555.2	7	47.62	37.8
OW964556.2	8	48.04	37.7
OW964557.2	9	47.04	37.7
OW964558.2	10	46.7	37.8
OW964559.2	11	46.23	37.7
OW964560.2	12	44.29	37.7
OW964561.2	13	45.48	37.8
OW964562.2	14	44.01	37.7
OW964563.2	15	44.33	37.8
OW964564.2	16	43.19	37.7
OW964565.2	17	42.57	37.8
OW964566.2	18	42.87	37.8
OW964567.2	19	41.98	37.7
OW964568.2	20	42.31	37.8
OW964569.2	21	41.18	37.6
OW964570.2	22	38.34	38
OW964571.2	23	42.05	37.7
OW964572.2	24	37.87	38
OW964573.2	25	40.27	37.7
OW964574.2	26	30.36	38.3
OW964575.2	27	30.46	38.1
OW964576.2	28	29.18	38
OW964577.2	29	29.08	37.8
OW964578.2	30	27.34	38.1
OW964548.2	Z	55.9	37.6
OW964579.2	MT	0.02	18.8
-	unplaced	5.23	41.3

Metadata for specimens, spectral estimates, sequencing runs, contaminants and pre-curation assembly statistics can be found at https://links.tol.sanger.ac.uk/species/988041.

### Genome annotation report

The *T. decimalis* assembly GCA_943138885.1 genome was annotated using the Ensembl rapid annotation pipeline ( Table 1; https://rapid.ensembl.org/Tholera_decimalis_GCA_943138885.1/Info/Index). The resulting annotation includes 22,825 transcribed mRNAs from 12,771 protein-coding and 1,968 non-coding genes.

## Methods

### Sample acquisition and nucleic acid extraction

A male *Tholera decimalis* (ilThoDeci1) was collected from Wytham Woods, Oxfordshire (biological vice-county: Berkshire), UK (latitude 51.77, longitude –1.34) on 8 September 2020. The specimen was taken from woodland habitat by Douglas Boyes (University of Oxford) using a light trap. The specimen was identified by the collector and snap-frozen on dry ice.

DNA was extracted at the Tree of Life laboratory, Wellcome Sanger Institute (WSI). The ilThoDeci1 sample was weighed and dissected on dry ice with tissue set aside for Hi-C sequencing. Head and thorax tissue was cryogenically disrupted to a fine powder using a Covaris cryoPREP Automated Dry Pulveriser, receiving multiple impacts. High molecular weight (HMW) DNA was extracted using the Qiagen MagAttract HMW DNA extraction kit. Low molecular weight DNA was removed from a 20 ng aliquot of extracted DNA using the 0.8X AMpure XP purification kit prior to 10X Chromium sequencing; a minimum of 50 ng DNA was submitted for 10X sequencing. HMW DNA was sheared into an average fragment size of 12–20 kb in a Megaruptor 3 system with speed setting 30. Sheared DNA was purified by solid-phase reversible immobilisation using AMPure PB beads with a 1.8X ratio of beads to sample to remove the shorter fragments and concentrate the DNA sample. The concentration of the sheared and purified DNA was assessed using a Nanodrop spectrophotometer and Qubit Fluorometer and Qubit dsDNA High Sensitivity Assay kit. Fragment size distribution was evaluated by running the sample on the FemtoPulse system.

RNA was extracted from abdomen tissue of ilThoDeci1 in the Tree of Life Laboratory at the WSI using TRIzol, according to the manufacturer’s instructions. RNA was then eluted in 50 μl RNAse-free water and its concentration assessed using a Nanodrop spectrophotometer and Qubit Fluorometer using the Qubit RNA Broad-Range (BR) Assay kit. Analysis of the integrity of the RNA was done using Agilent RNA 6000 Pico Kit and Eukaryotic Total RNA assay.

### Sequencing

Pacific Biosciences HiFi circular consensus and 10X Genomics read cloud DNA sequencing libraries were constructed according to the manufacturers’ instructions. DNA sequencing was performed by the Scientific Operations core at the WSI on Pacific Biosciences SEQUEL II (HiFi), Illumina HiSeq 4000 (RNA-Seq) and Illumina NovaSeq 6000 (10X) instruments. Hi-C data were also generated from head tissue of ilThoDeci1 using the Arima2 kit and sequenced on the Illumina NovaSeq 6000 instrument.

### Genome assembly, curation and evaluation

Assembly was carried out with Hifiasm ( Cheng *et al.*, 2021) and haplotypic duplication was identified and removed with purge_dups ( Guan *et al.*, 2020). One round of polishing was performed by aligning 10X Genomics read data to the assembly with Long Ranger ALIGN, calling variants with FreeBayes ( Garrison & Marth, 2012). The assembly was then scaffolded with Hi-C data ( Rao *et al.*, 2014) using YaHS ( Zhou *et al.,* 2023). The assembly was checked for contamination as described previously ( Howe *et al.*, 2021). Manual curation was performed using HiGlass ( Kerpedjiev *et al.*, 2018) and Pretext ( Harry, 2022). The mitochondrial genome was assembled using MitoHiFi ( Uliano-Silva *et al.*, 2022), which runs MitoFinder ( Allio *et al.*, 2020) or MITOS ( Bernt *et al.*, 2013) and uses these annotations to select the final mitochondrial contig and to ensure the general quality of the sequence. To evaluate the assembly, MerquryFK was used to estimate consensus quality (QV) scores and *k*-mer completeness ( Rhie *et al.*, 2020). The genome was analysed within the BlobToolKit environment ( Challis *et al.*, 2020) and BUSCO scores ( Manni *et al.*, 2021; Simão *et al.*, 2015) were calculated. Table 3 contains a list of software tool versions and sources.

**Table 3.** Software tools: versions and sources.

Software tool	Version	Source
BlobToolKit	4.0.7	https://github.com/blobtoolkit/blobtoolkit
BUSCO	5.3.2	https://gitlab.com/ezlab/busco
FreeBayes	1.3.1-17-gaa2ace8	https://github.com/freebayes/freebayes
Hifiasm	0.16.1-r375	https://github.com/chhylp123/hifiasm
HiGlass	1.11.6	https://github.com/higlass/higlass
Long Ranger ALIGN	2.2.2	https://support.10xgenomics.com/genome-exome/ software/pipelines/latest/advanced/other-pipelines
Merqury	MerquryFK	https://github.com/thegenemyers/MERQURY.FK
MitoHiFi	2	https://github.com/marcelauliano/MitoHiFi
PretextView	0.2	https://github.com/wtsi-hpag/PretextView
purge_dups	1.2.3	https://github.com/dfguan/purge_dups
YaHS	yahs-1.1.91eebc2	https://github.com/c-zhou/yahs

### Genome annotation

The Ensembl gene annotation system ( Aken *et al.*, 2016) was used to generate annotation for the *Tholera decimalis* assembly (GCA_943138885.1). Annotation was created primarily through alignment of transcriptomic data to the genome, with gap filling via protein-to-genome alignments of a select set of proteins from UniProt ( UniProt Consortium, 2019).

### Ethics and compliance issues

The materials that have contributed to this genome note have been supplied by a Darwin Tree of Life Partner. The submission of materials by a Darwin Tree of Life Partner is subject to the Darwin Tree of Life Project Sampling Code of Practice. By agreeing with and signing up to the Sampling Code of Practice, the Darwin Tree of Life Partner agrees they will meet the legal and ethical requirements and standards set out within this document in respect of all samples acquired for, and supplied to, the Darwin Tree of Life Project. All efforts are undertaken to minimise the suffering of animals used for sequencing. Each transfer of samples is further undertaken according to a Research Collaboration Agreement or Material Transfer Agreement entered into by the Darwin Tree of Life Partner, Genome Research Limited (operating as the Wellcome Sanger Institute), and in some circumstances other Darwin Tree of Life collaborators.

## Data Availability

European Nucleotide Archive: *Tholera decimalis* (feathered gothic). Accession number PRJEB52581; https://identifiers.org/ena.embl/PRJEB52581. ( Wellcome Sanger Institute, 2022) The genome sequence is released openly for reuse. The *Tholera decimalis* genome sequencing initiative is part of the Darwin Tree of Life (DToL) project. All raw sequence data and the assembly have been deposited in INSDC databases. Raw data and assembly accession identifiers are reported in Table 1.

## Author information

Members of the University of Oxford and Wytham Woods Genome Acquisition Lab are listed here: https://doi.org/10.5281/zenodo.4789928.

Members of the Darwin Tree of Life Barcoding collective are listed here: https://doi.org/10.5281/zenodo.4893703.

Members of the Wellcome Sanger Institute Tree of Life programme are listed here: https://doi.org/10.5281/zenodo.4783585.

Members of Wellcome Sanger Institute Scientific Operations: DNA Pipelines collective are listed here: https://doi.org/10.5281/zenodo.4790455.

Members of the Tree of Life Core Informatics collective are listed here: https://doi.org/10.5281/zenodo.5013541.

Members of the Darwin Tree of Life Consortium are listed here: https://doi.org/10.5281/zenodo.4783558.
