# Supplementary material for: The genome sequence of the Feathered Gothic, Tholera decimalis (Poda, 1761)
Source: Wellcome Open Res. 2023 May 5;8:200. [Version 1] doi: 10.12688/wellcomeopenres.19395.1 (PMC10477738; doi:10.12688/wellcomeopenres.19395.1)
Supplement: Peer review history

# Reviewer report: Reviewer response for version 1

**DOI:** 10.21956/wellcomeopenres.21483.r66106

**Published:** 2023-09-22

**Authors:** Arun Sethuraman, Priyanshi Shah

**Affiliations:** 1 San Diego State University, San Diego, California, USA; 2 Biology, San Diego State University, San Diego, California, USA

In this well-written manuscript, Boyes et al., describe the genome sequencing, assembly, and preliminary annotation of a single female *Tholera decimalis*. By assessing the assembly using the 3C criterion (contiguity, completeness, and correctness), they present a highly contiguous assembly, with a 99% score on the BUSCO v5.3.2 completeness, with minimal duplicated sequences. The authors have also assembled the Z sex chromosome and mitochondrial genome. There's a logical flow between sections and paragraphs where figures and tables are appropriately labeled and align with the text. The authors have also appropriately addressed ethical issues. We found the manuscript itself to be very informative, with sufficient detail. The genome (when released) will be a great utility to the Lepidopteran research community. We however had just few suggestions/comments that would help improve this resource:

1. While the assembly, annotation, and quality assessment methods are well described, it would be of great help to the community if the authors were to share their code/scripts/pipeline via a GitHub/Zenodo page. This would also ensure reproducibility of the methods. 
2. We were also unable to access the assembly via the Darwin Tree of Life portal (perhaps because of an embargo on release). It would be very helpful to make sure that the resource is released.
3. On page 3, under methods (sample acquisition and nucleic acid extraction), it’s mentioned that a “male” *Tholera decimalis* was collected when it was actually a female? Please clarify this.

Are sufficient details of methods and materials provided to allow replication by others?

No

Is the rationale for creating the dataset(s) clearly described?

Yes

Are the datasets clearly presented in a useable and accessible format?

Partly

Are the protocols appropriate and is the work technically sound?

Yes

Reviewer Expertise:

Population genomics, genomics, evolution

We confirm that we have read this submission and believe that we have an appropriate level of expertise to confirm that it is of an acceptable scientific standard, however we have significant reservations, as outlined above.

# Reviewer report: Reviewer response for version 1

**DOI:** 10.21956/wellcomeopenres.21483.r66112

**Published:** 2023-09-04

**Authors:** Jacqueline Heckenhauer

**Affiliations:** 1 Senckenberg Research Institute and Natural History Museum Frankfurt, Frankfurt, Germany

In their data note, the authors present ****the genome sequence of the primary assembly and contigs of the alternate haplotype of *Tholera decimalis *using a *de novo* assembly method following the approach used by the Darwin Tree of Life Project. The presented genome is of high quality and released openly for reuse. Therefore this high-quality reference genome is very beneficial to the field.

 However, I suggest that the authors include a short summary of repetitive DNA components of the assembly and submit the library of repetitive element (RE) sequences to a public repository of repetitive elements (e.g., Dfam). This way the community would even more benefit from this data. The diversity of available insect genomes has rapidly expanded, but the rate of community contributions to RE databases which are important for RE annotation has not kept pace, preventing high-resolution study of REs in many groups. 

 Was the genome size of this specimen / species estimated by flow cytometry or similar?

Are sufficient details of methods and materials provided to allow replication by others?

Yes

Is the rationale for creating the dataset(s) clearly described?

Yes

Are the datasets clearly presented in a useable and accessible format?

Yes

Are the protocols appropriate and is the work technically sound?

Yes

Reviewer Expertise:

evoultion, biodiversity genomics

I confirm that I have read this submission and believe that I have an appropriate level of expertise to confirm that it is of an acceptable scientific standard.

# Reviewer report: Reviewer response for version 1

**DOI:** 10.21956/wellcomeopenres.21483.r57063

**Published:** 2023-05-23

**Authors:** Hidemasa Bono

**Affiliations:** 1 Genome Editing Innovation Center, Hiroshima University, Higashi-Hiroshima, Hiroshima, Japan; 2 Graduate School of Integrated Sciences for Life, Hiroshima University, Higashi-Hiroshima, Hiroshima, Japan; 3 Database Center for Life Science, Research Organization of Information and Systems, Mishima, Shizuoka, Japan

Authors present the genome sequencing of the feathered gothic *Theolera decimalis. *The reads used in this study were sufficiently deep (45-fold coverage in PacBio HiFi) with Hi-C reads. The assembly constructed was very good judged from BUSCO v5.3.2 completeness score (99.0%). The computational programs used in that process were clearly summarized in Table 3 with precise version information. Reviewer has no more requests on the manuscript as a genome sequencing report.

Are sufficient details of methods and materials provided to allow replication by others?

Yes

Is the rationale for creating the dataset(s) clearly described?

Yes

Are the datasets clearly presented in a useable and accessible format?

Yes

Are the protocols appropriate and is the work technically sound?

Yes

Reviewer Expertise:

Genome informatics for non-model organisms

I confirm that I have read this submission and believe that I have an appropriate level of expertise to confirm that it is of an acceptable scientific standard.
